# Virtual Reality-Based Therapy Improves Balance, Quality of Life, and Mitigates Pain and Fear of Falling in Women with Bone Mineral Density Loss: A Meta-Analysis of Randomized Controlled Trials

**DOI:** 10.3390/life15111654

**Published:** 2025-10-23

**Authors:** Irene Cortés-Pérez, Ángeles Díaz-Fernández, María Catalina Osuna-Pérez, Héctor García-López, Raúl Romero-Del-Rey, Esteban Obrero-Gaitán

**Affiliations:** 1 Department of Health Sciences, University of Jaén, Campus Las Lagunillas s/n, 23071 Jaén, Spain; icortes@ujaen.es (I.C.-P.); mcosuna@ujaen.es (M.C.O.-P.); 2 Department of Nursing, Physiotherapy and Medicine, University of Almería, Ctra. Sacramento s/n, La Cañada, 04120 Almería, Spain; hector.garcia@ual.es (H.G.-L.); rrd239@ual.es (R.R.-D.-R.)

**Keywords:** bone mineral density loss, osteoporosis, sarcopenia, women, virtual reality, postural balance, pain, quality of life

## Abstract

**Background/Objectives**: Performing therapeutic exercises using video games via virtual reality devices can be effective for preventing or mitigating bone mineral density (BMD) loss. The objective of this paper is to determine the effectiveness of virtual reality-based therapy (VRBT) in improving BMD, postural balance, fear of falling, pain intensity, and quality of life (QoL) in women with BMD loss. **Methods**: A systematic review with meta-analysis (SRMA), after searching in PubMed Medline, WOS, Scopus, CINAHL, and PEDro up to July 2025, was conducted following PRISMA guidelines. Randomized controlled trials (RCTs), including women with BMD loss, in which VRBT was compared to conventional approaches for the management of BMD loss, were included. Methodological quality and quality of evidence were assessed using the PEDro scale and the GRADE statement, respectively. Effect size was estimated through standardized mean difference (SMD) and 95% confidence interval (95% CI). **Results**: Seven RCTs, involving 299 women with BMD loss (mean age: 60.5 ± 7.7 years), were included in this SRMA. All VRBT employed non-immersive virtual reality (NIVR) devices.Significant effectiveness on BMD loss (SMD = 0.92; *p* = 0.002), functional (SMD = 1.7; *p* < 0.001) and dynamic balance or functional mobility (SMD = −1.7; *p* = 0.01), fear of falling (SMD = −0.5; *p* = 0.009), pain intensity (SMD = −2.7; *p* = 0.039) and QoL (SMD = 3.9; *p* = 0.002) was reported favors VRBT in women with BMD loss. **Conclusions**: This SRMA demonstrates that VRBT (especially NIVR) can be effective at improving BMD, postural balance, functional mobility, and QoL, while simultaneously reducing pain intensity and the fear of falling in these women.

## 1. Introduction

Bone fragility, encompassing pathologies such as osteopenia and osteoporosis, represents a growing global public health challenge, especially among the female population [1]. These conditions, characterized by a progressive decrease in bone mineral density (BMD) and a deterioration of skeletal tissue microarchitecture, predispose women to a higher risk of fractures, even from minor traumas [2]. BMD loss not only impacts the structural integrity of the skeleton but also triggers a series of consequences that reduces quality of life (QoL) [3]. BMD is related firstly to osteopenia and progressively to osteoporosis. On the one hand, osteopenia is defined by a BMD that is lower than normal but not low enough to be classified as osteoporosis [4]. Although often asymptomatic, osteopenia signals an early warning of unfavorable bone metabolism and an increased risk of progression to osteoporosis over time [5]. Osteoporosis, on the other hand, stands as a systemic skeletal disease characterized by pronounced BMD loss and increased bone porosity, compromising bone strength and multiplying susceptibility to fractures [6,7]. These fractures not only cause acute and chronic pain but also lead to high morbidity, long-term disability, decreased functional independence, and in some cases, increased mortality [8]. BMD loss results from an imbalance between bone resorption and formation. Under physiological conditions, these processes are regulated to maintain skeletal homeostasis. However, in osteopenia and osteoporosis, bone resorption outpaces formation, leading to a net decrease in bone mass and a deterioration of bone tissue quality [9]. Hormonal factors, such as decreased estrogen levels during menopause, play a crucial role in accelerating bone loss in women. Other risk factors include advanced age, family history, low body weight, calcium and vitamin D deficiency, smoking, and excessive alcohol consumption [10].

The BMD loss has become a global public trend due to the severity of physical, psychological, functional, and social disabilities and their impacts on these women. Women with BMD loss report reduced physical mobility and functional capability related to daily living activities (DLAs). BMD loss can severely impact a person’s ability to maintain balance, often resulting in a disabling condition. The associated muscle weakness (especially in leg and trunk muscles) [11,12] and alterations in proprioception and visual function related to aging increase the likelihood of trips and loss of balance [13,14], also contributing to an increased risk of falls [15]. The risk of falls is a primary concern in patients with LBMD [16]. The consequences of falls in these women can be devastating, resulting in fractures that require hospitalization, surgery, and long periods of rehabilitation [17]. Even falls without fracture can generate fear of falling in the future and reduce confidence in their own DLA-related balance [18,19]. Additionally, pain, especially after a fracture, can also debilitate and significantly affect the ability to perform DLAs [20]. Recent studies have demonstrated a positive association between osteoporosis and worse QoL, comparable to that of other severe chronic diseases [21,22]. The low self-esteem and functional status, anxiety, and/or depression due to this disabling vicious cycle negatively impact overall well-being [23], limiting participation in DLAs and reducing QoL [24]. For reducing the impact of these disabling consequences of BMD loss, it is crucial to find effective and safe interventions.

Traditionally, the most common interventions for the management of BMD are pharmacotherapy (mainly calcium and vitamin D supplementation) and physical exercise programs for increasing muscle strength and balance skills [25,26]. However, long-term adherence to these treatments can be a challenge, and the need for complementary, innovative, and safe approaches is evident [27,28]. According to this, virtual reality-based therapy (VRBT) emerged as a promising tool for this purpose. Virtual reality (VR) devices are characterized by sophisticated software and hardware that reproduce a virtual environment that users feel to be real [29]. VR devices can be categorized into three different modalities: Non-immersive virtual reality (NIVR), semi-immersive VR, and immersive VR [30]. NIVR is the modality most used in clinical practice related to musculoskeletal and balance disorders and can offer several potential advantages for addressing the challenges associated with osteopenia and osteoporosis [31,32]. NIVR utilizes computer screens, televisions, or projectors to display interactive virtual environments. Users interact with these environments through peripherals such as keyboards, mice, game controllers, or motion sensors [33].

In addition to the growing interest in VRBT as a therapeutic tool in musculoskeletal conditions [34], in the management of osteopenia and osteoporosis, it has also been shown to be effective [35]. Specifically, regarding these conditions, no previous reviews have exclusively assessed the effectiveness of VRBT on BMD loss. Thuilier, E et al. (2024) conducted a heterogeneous systematic review including older adults with musculoskeletal conditions in which virtual and augmented reality effects were assessed [36]. In 2025, He et al. conducted a meta-analysis including five studies where they suggested that VRBT could be effective in increasing BMD and balance, but not QoL [37]. These reviews present common limitations related to language filters in the literature search (excluding studies not in English or French). Additionally, the very low number of studies included in the meta-analyses of He et al. (2005) [37] does not allow us to generalize the findings, requiring a new and more concise systematic review, retrieving more studies. Therefore, the aim of this systematic review with meta-analysis (SRMA) was to assess the effectiveness of VRBT in improving BMD, balance, fear of falling, pain intensity, and QoL in women with BMD loss.

## 2. Materials and Methods

### 2.1. Study Design

Authors followed PRISMA 2020 guidelines [38] and the Cochrane Handbook for Systematic Reviews of Interventions recommendations [39]. The AMSTAR 2 checklist evaluated the methodological quality of this SRMA [40]. This SRMA has been previously registered in PROSPERO (CRD420251057144).

### 2.2. Data Sources

The literature search process was conducted exhaustively by two independent authors (I.C.-P. and E.O.-G.) from inception up to July 2025, without publication date or language limitations, in PubMed Medline, WOS, Scopus, CINAHL, and PEDro. Additionally, authors screened the reference lists of previous studies, including congress abstracts and proceedings. The PICOS framework was followed to define the search strategy [41]: population (women with LBMD), intervention (VRBT), comparison (conventional exercise therapy), outcomes (BMD, postural balance, fear of falling, pain, and QoL), and study design (randomized controlled trials [RCTs] or pilot RCTs). Sensitivity in search strategy was increased by combining only terms related to the population and intervention with the Boolean operators “AND” and “OR”. Keywords combined in the search, according to the PubMed thesaurus, were “osteoporosis”, “osteoporosis postmenopausal”, “virtual reality”, and “exergaming” (Table 1).

### 2.3. Study Selection: Inclusion and Exclusion Criteria

Two authors (A.D.-F. and M.C.O.-P.) independently conducted a screening of studies retrieved by title and abstract. Inter-rater agreement was estimated using the Cohen’s kappa coefficient (κ) [42], with κ values interpreted as follows: null (κ = 0), low (0 < κ ≤ 0.20), medium (0.41 ≤ κ ≤ 0.60), or excellent (0.81 ≤ κ ≤ 1) [43]. Disagreements were resolved by a third researcher (I.C.-P.) Authors included studies that met the PICOS items (named in the previous section). As additional inclusion criteria, authors reported that the RCTs included must provide statistical data (mean and standard deviation) of the variables of interest to conduct the meta-analysis. Authors excluded studies that included patients with and without BMD loss in the same sample.

### 2.4. Data Extraction

Two authors (A.D.-F. and M.C.O.-P.) independently compiled data from the studies selected in a data collection form with the participation of a third author (I.C.-P.) in resolving doubts according to this process. The data extracted can be categorized as (1) overall characteristics (authorship, date of publication, country, blinding status, setting, and financing); (2) characteristics of the participants (total sample size and sample size per group, age, sex, and diagnosis of BMD loss); (3) VRBT characteristics (software and modality of VRBT employed, as well as protocol of application in total number of sessions, weeks, and time of use of VR in each session); (4) comparison intervention (type of comparison and protocol of application); and (5) variables (name, method of evaluation, and statistics data needed for meta-analysis [mean and standard deviation]).

### 2.5. Variables

BMD is defined in clinical practice as the amount of mineral per square centimeter of bone. Postural balance is categorized as functional (ability to maintain balance during functional activities) and dynamic balance (skill to maintain standing and stability during movement or displacement); fear of falling; pain; and QoL.

### 2.6. Methodological Quality, Risk of Bias, and Quality of Evidence Assessment

On the one hand, the PEDro Scale, an 11-item scale designed to evaluate the methodological quality in RCTs [44], was used to assess the methodological quality of the studies included. Methodological quality was categorized as follows: excellent (10–9 points), good (8–6 points), moderate (5–4 points), and poor (≤3 points) [45]. Related to identifying potential biases in RCTS using the PEDro scale, selection, performance, and detection biases can be identified through items 2–3, 5–6, and 7, respectively.

On the other hand, the level of evidence for each outcome was determined through the GRADE assessment and Meader’s GRADE checklist [46,47], considering if the following items (risk of bias, inconsistency, imprecision, indirectness, and risk of publication bias) are met. The level of evidence, which could be categorized as high, moderate, low, or very low, was downgraded for each item not met.

These assessments were conducted by two authors (H.G.-L. and R.R.-R.), independently, supervised by a third author (E.O.-G.).

### 2.7. Statistical Analysis

The meta-analysis was conducted by two authors (I.C.-P. and E.O.-G.) using Comprehensive Meta-Analysis software version 4 (Biostat, Englewood, NJ, USA) [48]. The Cohen’s standardized mean difference (SMD) and 95% confidence intervals (95% CI) were used as the effect size measure in a random-effects model [49,50]. According to Kinney et al. (2020), the effect size was interpreted as null (SMD = 0), low (SMD = 0.08 − 0.15), medium (SMD = 0.19 − 0.36), and large (SMD > 0.4) [51]. The findings were displayed in the forest plots [52]. Publication bias was estimated through the funnel plot visualization, Egger’s test, and the trim-and-fill calculation [53,54,55]. If the difference between the original and adjusted pooled effect size exceeded 10%, the level of evidence was downgraded by one level when the difference between the effect size and adjusted effect size was greater than 10% [56]. The degree of inconsistency of Higgins (I^2^), the χ-square test, and its *p*-value were considered to calculate the inconsistency or heterogeneity [57,58], which can be large (I^2^ > 50%), moderate (I^2^ 25–50%), low (I^2^ 5–25%), or null (I^2^ ≤ 5%) [59]. Additionally, the contribution of the studies to the global effect size was estimated by applying the leave-one-out method.

## 3. Results

### 3.1. Study Selection

**Figure 1:**
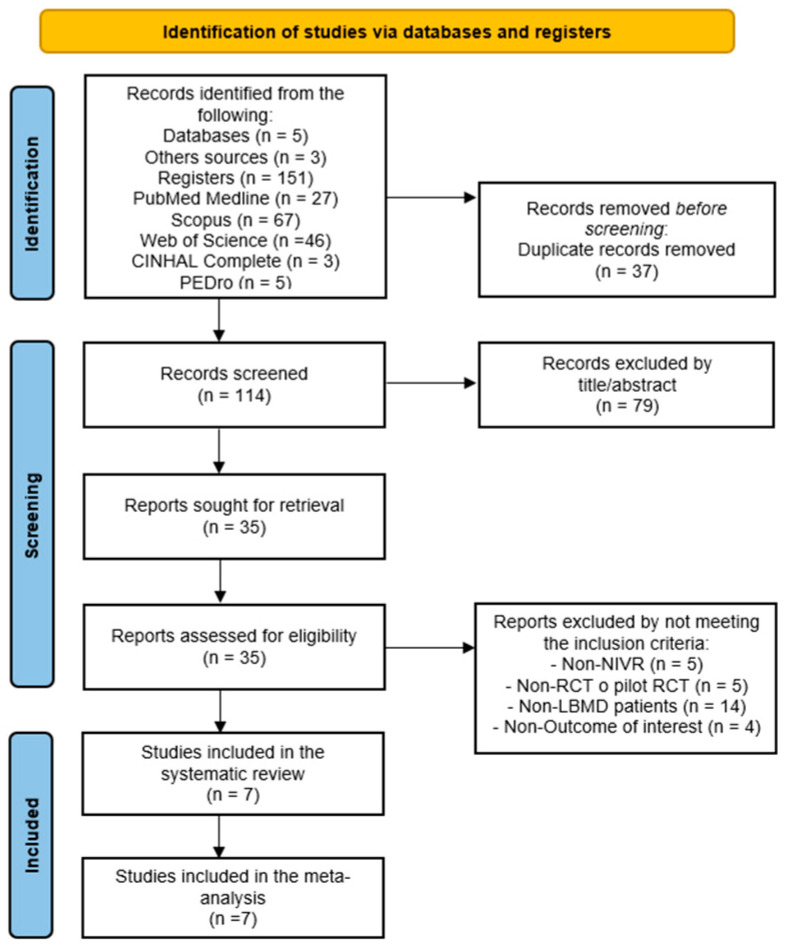
PRISMA flow diagram.

A total of 154 records were retrieved, 151 from databases (PubMed Medline n = 27; Scopus n = 67; WOS n = 46; CINAHL n = 3; and PEDro n = 5), and 3 references were from others. After removing 37 duplicates, 114 references were examined by title and abstract, of which 79 references were removed for not being relevant. In total, 28 studies did not meet the inclusion criteria (non-RCT or non-pilot RCT n = 5, non-VRBT n = 5, non-BMD loss patients n = 14, and non-outcomes of interest n = 4). Finally, this SRMA included seven RCTs [60,61,62,63,64,65,66]. The authors reported an excellent inter-rater agreement (κ = 0.94) in this stage. Figure 1 shows the PRISMA flow diagram for study selection.

### 3.2. Characteristics of the Studies Included

The included RCTs, conducted between 2016 and 2024 in Iran, Italy, Saudi Arabia, Pakistan, Turkey, and China, enrolled 299 women with BMD loss (mean age: 60.5 ± 7.7 years). The intervention group included 143 women who received VRBT. All VRBT interventions conducted by the RCTs included were NIVR, ranging from 6 to 48 weeks, including sessions from 16 to 144 sessions, lasting between 45 and 60 min per session. VRBT interventions included strength, balance, and aerobic exercises using NIVR headsets such as the Nintendo Wii, Xbox Kinect, Pro-Kin system, and VR rehabilitation system. The comparison group included 156 women who received conventional training, such as strength exercises, balance, aerobics, and walking outdoors. All RCTs only provided post-intervention data. Four RCTs reported receiving grants to carry out the study [60,63,64,65]. Table 2 summarizes the characteristics of these studies.

### 3.3. Methodological Quality and Risk of Bias

The included RCTs exhibited good methodological quality (6.6 ± 0.5 points in PEDro). The score of four RCTs had been previously confirmed on the PEDro database [60,63,64,66]. All RCTs ranged from 6 to 7 points. Selection bias was present in two RCTs due to inadequate concealed allocation (item 3 was not met) [63,64]. All RCTs reported risk of performance bias, due to the inadequate blinding of participants and therapists (items 5 and 6 were not met). Finally, detection bias, due to evaluators not being blinded (item 7 not met), was present in two RCTs [63,64]. Table 3 shows the PEDro assessment of each RCT included in this SRMA.

### 3.4. Meta-Analysis

#### 3.4.1. Bone Mineral Density

**Figure 2:**
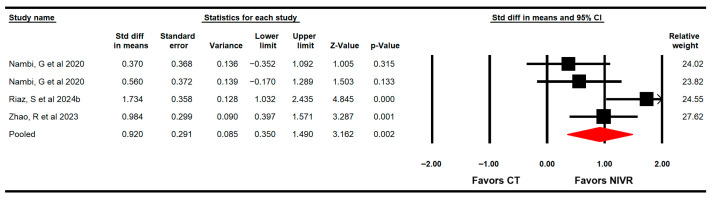
Forest plot of the effectiveness of virtual reality-based interventions on bone mineral density [64,65,66].

The effectiveness of VRBT in increasing the BMD was assessed through three RCTS with four independent comparisons [64,65,66]. These studies provided data from 153 women (38.3 per study) using dual-energy X-ray absorptiometry. The meta-analysis exhibited that VRBT is largely effective (SMD = 0.92; 95% CI 0.35 to 1.5; *p* = 0.002; I2 = 4.9%; χ^2^ = 3.1; df = 3; *p* = 0.38) in increasing the BMD in these women (Figure 2). Risk of publication was not reported (Egger *p* = 0.78).

#### 3.4.2. Functional Balance

**Figure 3:**
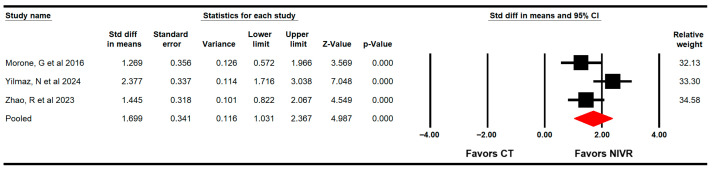
Forest plot of the effectiveness of virtual reality-based interventions on functional balance [61,63,64].

Three RCTs with three independent comparisons [61,63,64] provided data from 148 women (49.3 per study) to assess the effectiveness of VRBT on functional balance through the Berg Balance Scale (BBS). The meta-analysis showed a large effect (SMD = 1.7; 95% CI 1.03 to 2.36; *p* < 0.001; I2 = 1%; χ^2^ = 2.1; df = 2; *p* = 0.36), favoring VRBT in increasing functional balance (Figure 3) without risk of publication bias (Egger *p* = 0.47).

#### 3.4.3. Dynamic Balance/Functional Mobility

**Figure 4:**
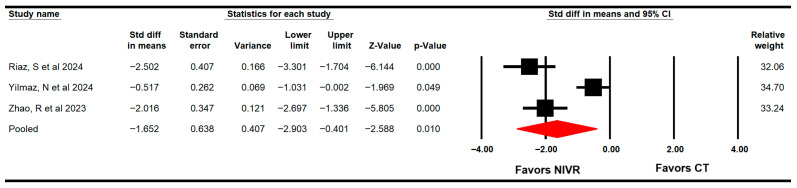
Forest plot of the effectiveness of virtual reality-based interventions on dynamic balance and functional mobility [62,63,64].

Three RCTs with three independent comparisons [62,63,64] that provided data from 153 participants (51 per study) using the Timed Up and Go Test (TUG) were included to assess the effect of VRBT on dynamic balance or functional mobility. The TUG test is a measurement used to assess both dynamic balance and functional mobility [67]. Our findings showed that VRBT is largely effective (SMD = −1.7; 95% CI −2.9 to −0.4; *p* = 0.01; I2 = 0%; χ^2^ = 1.8; df = 2; *p* = 0.41) in increasing dynamic balance and functional mobility (Figure 4) without risk of publication bias (Egger *p* = 0.19).

#### 3.4.4. Fear of Falling

**Figure 5:**
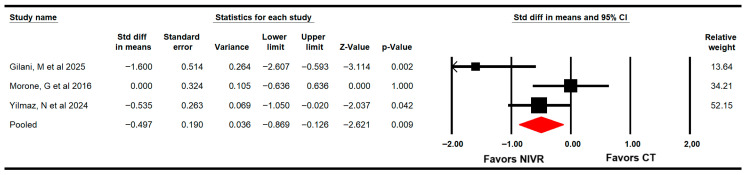
Forest plot of the effectiveness of virtual reality-based interventions on fear of falling [60,61,63].

The effectiveness of VRBT in reducing the fear of falling was assessed through three RCTs with three independent comparisons [60,61,63]. These studies provided data from 118 women (39.3 per study) using the Falls Efficacy Scale (FES). The meta-analysis determined that VRBT is largely effective (SMD = −0.5; 95% CI −0.9 to −0.13; *p* = 0.009; I2 = 29%; χ^2^ = 2.7; df = 2; *p* = 0.3) in reducing the fear of falling in these patients (Figure 5). No risk of publication was found (Egger *p* = 0.21).

#### 3.4.5. Pain

**Figure 6:**
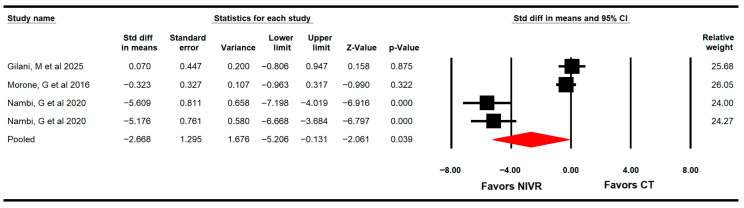
Forest plot of the effectiveness of virtual reality-based interventions on pain intensity [60,61,65].

Three RCTs with four independent comparisons [60,61,65] provided data from 118 women (29.5 per study) to assess the effectiveness of VRBT in alleviating pain intensity through the Visual Analog Scale (VAS), Qualeffo-41, and the Quality of Life questionnaire for pain. The meta-analysis showed a large effect (SMD = −2.7; 95% CI −5.2 to −0.13; *p* = 0.039 I2 = 27.1%; χ^2^ = 4.2; df = 3; *p* = 0.24), favoring VRBT in reducing pain (Figure 6). These findings were influenced by the risk of publication bias (Egger *p* = 0.03), confirmed using the trim-and-fill estimation that showed that the risk of publication bias could be overestimating 60% of the original pooled effect of NIVR (adjusted SMD = −1.6).

#### 3.4.6. Quality of Life

**Figure 7:**
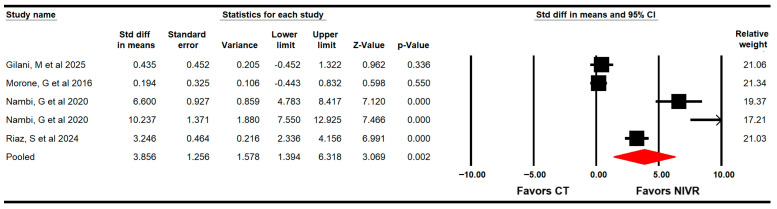
Forest plot of the effectiveness of virtual reality-based interventions on quality of life [60,61,62,65].

Four RCTs with five independent comparisons [60,61,62,65] provided data from 103 women (20.6 per study) to assess the effectiveness of VRBT in improving QoL through the Qualeffo-41, SF-36, and Quality of Life questionnaire. The meta-analysis showed a large effect (SMD = 3.9; 95% CI 1.4 to 6.3; *p* = 0.002; I2 = 44.7%; χ^2^ = 8.8; df = 4; *p* = 0.06), favoring NIVR in increasing QOL (Figure 7). No risk of publication was found (Egger *p* = 0.11).

## 4. Discussion

To our knowledge, this is the first SRMA to retrieve the largest number of RCTs available to date, specifically focused on VRBT through NIVR devices, in women with BMD loss, which adds originality and clinical relevance to the previous findings [36,37]. Due to the lack of previous reviews that assessed the effect of VRBT for the management of BMD loss in women, the objective of our SRMA was to assess it, including the largest number of RCTs available to date. Therefore, this SRMA retrieved seven RCTs [60,61,62,63,64,65,66] that provided data from 299 women with BMD loss that compared VRBT with traditional approaches for the management of clinical and functional outcomes in women with BMD loss. All RCTs conducted VRBT with NIVR. The studies reviewed varied in duration (6 to 48 weeks), frequency (2 to 5 sessions per week), and devices used (Nintendo™ Wii, Xbox™ Kinect, Pro-Kin, or custom VR platforms), but all implemented interventions were based on movement simulation, visual feedback, and task-oriented exercises. The findings support the potential of NIVR to increase BMD, postural balance, functional mobility, and QoL, as well as reduce the fear of falling and pain in these women, with varying degrees of evidence across domains. One strength of this SRMA is that it is the first to elucidate the use of VRBT in the management of BMD loss.

At first, our findings showed a significant effect of VRBT for increasing BMD in these women. This finding aligns with the meta-analysis by He et al., although their research only identified effectiveness for VRBT in increasing femoral neck BMD [37]. It points to the potential of NIVR devices to promote osteogenesis indirectly through improved compliance, increased movement variability, and longer total training exposure [36]. The RCTs included in this meta-analysis carried out physical, mobility, and walking exercises using NIVR devices [64,65,66], with physical exercise being one of the most important strategies to prevent the age-associated BMD loss [68]. The combination of physical exercises in virtual, gamified, and ludic environments can be responsible for this improvement in BMD.

Due to postural balance as a general concept, this SRMA assessed the effectiveness of VRBT in different balance domains (functional and dynamic). Our findings reported that VRBT is largely effective for increasing functional and dynamic balance as well as functional mobility in women with BMD loss. However, the low number of studies included in the meta-analysis of each outcome can reduce the generalization and precision of the findings. All VRBT included combined balance-specific exercises performed in dynamic contexts, suggesting that postural control can benefit from consistent exposure to VR-based multisensory stimulus (visual, auditory, and proprioceptive) necessary to maintain balance [69], promoting improved spatial orientation and dynamic stability [32,70,71]. While the magnitude of improvement varied, the convergence of outcomes suggests that NIVR enhances anticipatory postural adjustments and motor planning through repeated exposure to functional movement simulations [72,73]. Additionally, some video games used in NIVR devices, such as Nintendo™ Wii or Kinect™, reproduce real-world functional and ambulatory tasks, such as arm movement-oriented tasks or walking, whose repetitive practice can be responsible for the improvement in functional balance.

Consequently, regarding the improvement of balance and functional mobility, our SRMA also reported that the use of NIVR, as VRBT in these women, could largely reduce fear of falling. The confidence gains observed in the studies included in this SRMA may reflect not only physical improvements, but also cognitive reframing associated with repetitive success in virtual tasks. In particular, the studies by Morone et al. and Yilmaz et al., both using Wii-based training, involved tasks resembling real-life activities such as stepping, shifting weight, or reaching—scenarios that participants often perceive as threatening in daily life. The ability to practice these actions in a safe and controlled VR environment likely contributed to the psychological dimension of fall prevention [74,75].

Finally, we assessed the effectiveness of VRBT on pain and QoL. Our findings reported that VRBT was largely effective in reducing pain intensity and increasing QoL. On the one hand, related to pain, no previous SRMA has assessed this variable, highlighting the novelty of these findings. Additionally, the NIVR devices used combined balance and strength exercises with virtual feedback, which could have facilitated attentional diversion, movement confidence, and reduced guarding. Although the exact mechanisms remain speculative, it is plausible that repeated exposure to functional movement in a VR setting may contribute to neuroplastic adaptations in pain-processing circuits [76], a hypothesis that deserves further exploration in future studies. Nonetheless, these findings should be interpreted with caution, as the publication bias analysis indicated a potential overestimation of the pooled analgesic effect by approximately 60%, raising concerns about the robustness of this outcome. On the other hand, in relation to QoL, these findings disagree with the meta-analysis of He et al., who did not find statistically significant differences favoring VRBT in improving QoL [37]. Participants in NIVR groups experienced broad improvements in physical, emotional, and social well-being. Notably, the strongest effects were observed in studies that combined VR with walking or longer-duration interventions, such as in Riaz et al., where participants trained for 24 weeks. These results suggest that NIVR may influence biopsychosocial outcomes through multiple mechanisms—by increasing autonomy, decreasing isolation, and enhancing motivation to engage in physical activity [77].

Clinical implications include the dual capacity of NIVR to address both physical impairments (e.g., balance and strength) and psychosocial barriers (fear of falling and low motivation) by simulating real-life tasks within a controlled, engaging environment. This gamified approach not only delivers clinically meaningful improvements in mobility but also enhances treatment adherence and emotional well-being. Given the accessibility and affordability of many NIVR systems, including those based on commercial gaming platforms, these interventions are particularly well suited for integration into community health programs, outpatient clinics, and even home-based rehabilitation formats [78].

Although our findings are clinically relevant for improving BMD loss, several limitations should be considered. First, the analyses included a low number of RCTs with small sample sizes. This could negatively impact the quality of evidence and the generalizability of these novel findings. However, the search strategy employed allowed for the retrieval of all RCTs published to date, without language or publication date restrictions. Second, the presence of selection, performance, and detection biases in the included RCTs might affect the generalization of the findings and overestimate or underestimate the true effect of VRBT [79,80]. Third, the presence of risk of publication bias in meta-analysis concerning pain suggested that it overestimated the original effect, although it was corrected by applying the trim-and-fill calculation. While it is generally recommended to include at least 10 studies for a reliable assessment of publication bias, the limited number of RCTs conducted to date restricts the availability of such a quantity of studies in any meta-analysis. Furthermore, the heterogeneity among included RCTs regarding the characteristics of the VRBT may complicate inter-study comparisons, a common limitation in RCTs conducted in physiotherapy. Future studies should prioritize the adoption of stricter, standardized methodological designs. By expanding sample sizes and unifying intervention protocols and outcome measures, the field can ensure greater evidentiary reliability and thus better support evidence-based clinical practice.

## 5. Conclusions

This SRMA evaluates the effectiveness of VRBT (specifically NIVR) on women with BMD loss and is notable for including the largest number of RCTs published to date. Our findings elucidate that the integration of VRBT in rehabilitation programs for women with BMD loss increases BMD, balance, functional mobility, and QoL; it also reduces fear of falling and pain intensity compared to conventional therapies. These findings are of great clinical relevance to practitioners (including physiotherapists and nurses) because they highlight that the practice of different physical exercises in virtual, gamified, and ludic environments is appropriate for the management of BMD loss in these women. However, continued research is needed to clarify long-term effects and to optimize the parameters of these interventions for routine clinical use.

## Figures and Tables

**Table 1:** Search strategies in databases.

Database	Search Strategy
PubMed Medline	(Osteoporosis[mh] or osteoporosis[tiab] or low bone density[tiab] or osteoporosis postmenopausal[mh] or osteoporosis postmenopausal[tiab] or osteopenia[tiab]) and (virtual reality[mh] or virtual reality[tiab] or exergaming[mh] or exergame *[tiab] or virtual reality exposure therapy[mh] or virtual reality exposure therapy[tiab] or wii[tiab] or Nintendo[tiab] or videogame *[tiab] or non-immersive virtual reality[tiab] or kinect[tiab] or virtual environment[tiab] or seious game[tiab])
SCOPUS	(“Osteoporosis” or “low bone density” or “osteoporosis postmenopausal” or “osteopenia”) and (“virtual reality” or “exergaming” or “virtual reality exposure therapy” or “wii” or “Nintendo” or “videogamame” or “immersive virtual reality” or “Kinect” or “virtual environment” or “seious game”)
Web of Science	TOPIC(*Osteoporosis* or *low bone density* or *osteoporosis postmenopausal* or *osteopenia*) and TOPIC(*virtual reality* or *exergaming* or *virtual reality exposure therapy* or *wii* or *Nintendo* or *videogamame* or *immersive virtual reality* or *Kinect* or *virtual environment* or *seious game*)
CINAHL Complete	AB (Osteoporosis or low bone density or osteoporosis postmenopausal or osteopenia) and AB (virtual reality or exergaming or virtual reality exposure therapy or Wii or Nintendo or videogame or immersive virtual reality or kinect or virtual environment or serious game)
PEDro	Osteoporosis and virtual reality

**Table 2:** Characteristics of the studies included.

Study	Participants (N, Diagnosis, Age)	VR Group	Control group	Outcomes
		N, Characteristics of the Intervention	N, Characteristics of the Intervention	Variable (Test)
Gilani, M et al., 2023 (Iran) [60] Non-blinded RCT Setting: Physical Therapy Department at Tarbiat Modares University Funding: Yes	20 women diagnosed with primary osteoporosis Mean age: 58.9 ± 0.6 years	10 women performed strength and balance training in non-immersive virtual reality using the Xbox Kinect 360 system, 60 min per day, 3 days per week for 6 weeks.	10 women performed conventional training with balance and strength exercises, 60 min a day, 3 days a week for 6 weeks.	Fear of falling (FES-I) Quality of life (Qualeffo-41) Pain (Qualeffo-41)
Morone, G et al., 2016 (Italy) [61] Single-blinded RCT Setting: Physical Medicine and Rehabilitation, Policlinico Umberto I Funding: NR	38 women diagnosed with bone loss condition Mean age: 68.9 ± 1.6 years	19 women performed balance and strength training using the non-immersive virtual reality system Nintendo Wii, 60 min a day, 2 days a week for 8 weeks.	19 women performed conventional training with balance and strength exercises, 60 min a day, 2 days a week for 8 weeks.	Fear of falling (FES-I) Quality of life (SF-36) Pain (VAS) Functional balance (BBS)
Nambi, G et al., 2020 (Saudi Arabia) [65] Double-blinded RCT Setting: Physical Therapy Department Funding: Yes	45 women diagnosed with osteoporosis Mean age: 56.9 ± 0.9 years	15 women performed non-immersive virtual reality training with the Pro-Kin system for 45 min a day, 4 days a week, for 12 weeks. The training consisted of weight-bearing hip and knee mobility exercises.	Control group _1_: 15 women performed aerobic exercise training for 30 min, 4 days a week, for 12 weeks. Control group _2_: 15 women did not perform any specific exercise protocol for 12 weeks.	Quality of life (Quality of Life questionnaire) Pain (Quality of Life questionnaire) Bone mineral density (dual energy X-ray absorptiometry)
Riaz, S et al., 2024a (Pakistan) [64] Double-blinded RCT Setting: Riphah Rehabilitation Center Funding: No.	43 women diagnosed with osteopenia Mean age: 58.1 ± 0.2 years	22 women performed non-immersive virtual reality with Xbox Kinect, 45 min per session, 3 days a week for 24 weeks + outdoor walk 30 min per day, 7 days a week for 24 weeks.	21 women performed outdoor walks for 30 min a day, 7 days a week, for 24 weeks.	Quality of life (ECOS-16) Dynamic balance/mobility (TUG)
Riaz, S et al., 2024b (Pakistan) [66] Double-blinded RCT Setting: Riphah Rehabilitation Center Funding: No.	43 women diagnosed with osteopenia Mean age: 58.1 ± 0.2 years	22 women performed non-immersive virtual reality with Xbox Kinect, 45 min per session, 3 days a week for 24 weeks + outdoor walk 30 min per day, 7 days a week for 24 weeks.	21 women performed outdoor walks for 30 min a day, 7 days a week. for 24 weeks.	Bone mineral density (dual energy X-ray absorptiometry)
Yilmaz, N et al., 2024 (Turkey) [63] Non-blinded RCT Setting: NR. Funding: Yes.	60 women diagnosed with osteoporosis Mean age: 50 ± 4.1 years	30 women performed balance exercises with the non-immersive virtual reality system Nintendo Wii balance board, 45 min per session, 3 days a week for 12 weeks + walking and upper and lower limb mobility exercises as a warm-up.	30 women performed mobility, strengthening, stretching, and balance exercises, 45 min per session, 3 days a week for 12 weeks + walking and upper and lower limb exercises as a warm-up.	Fear of falling (FES-I) Functional balance (BBS) Dynamic balance/mobility (TUG)
Zhao, R et al., 2023 (China) [64] Nom-blinded RCT Setting: Elderly Healthcare Institution Funding: Yes.	50 women diagnosed with osteoporosis Mean age: 72.7 ± 0.8 years	25 women performed non-immersive virtual reality exercises with the VR rehabilitation training device, 50 min per session, 3 days a week for 12 months.	25 women performed an exercise routine to prevent falls, including aerobic and machine exercises, 50 min per session, 3 days a week for 12 months.	Functional balance (BBS) Dynamic balance/mobility (TUG) Bone mineral density (dual energy X-ray absorptiometry)

Abbreviations: RCT, Randomized Controlled Trial; NR, Non-Reported; FES-I, Falls Efficacy Scale International; VAS, Visual Analog Scale; TUG, Timed Up and Go Test; ECOS-16, Escala de la Calidad de Vida Osteoporosis; BBS, Berg Balance Scale.

**Table 3:** PEDro score of the studies included.

Study	Items											Total	Quality	Biases
	i1	i2	i3	i4	i5	i6	i7	i8	i9	i10	i11			
Gilani, M et al., 2023 * [60]	Y	Y	Y	Y	N	N	Y	Y	N	Y	Y	7/10	Good	Performance
Morone, G et al., 2016 [61]	Y	Y	Y	Y	N	N	Y	Y	N	Y	Y	7/10	Good	Performance
Nambi, G et al., 2020 [65]	Y	Y	Y	Y	Y	N	Y	N	N	Y	Y	7/10	Good	Performance
Riaz, S et al., 2024 * [64]	Y	Y	Y	Y	N	N	Y	N	N	Y	Y	6/10	Good	Performance
Riaz, S et al., 2024b [66]	Y	Y	Y	Y	Y	N	Y	N	N	Y	Y	7/10	Good	Performance
Yilmaz, N et al., 2024 * [63]	N	Y	N	Y	N	N	N	Y	Y	Y	Y	6/10	Good	Selection, performance and detection
Zhao, R et al., 2023 * [64]	Y	Y	N	Y	N	N	N	Y	Y	Y	Y	6/10	Good	Selection, performance and detection

Abbreviations: i1: Eligibility criteria; i2: Random allocation; i3: Concealed allocation; i4: Baseline comparability; i5: Blind subjects; i6: Blind therapists; i7: Blind assessors; i8. Measures of at least one key outcome were obtained from more than 85% of the subjects initially allocated to groups; i9: Intention-to-treat analysis; i10: Between-group comparisons; i11: Point estimates and variability; Y: Yes; N: No. Notes: The Eligibility criteria item does not contribute to the total score. The score of the studies marked with * was confirmed in the PEDro database (www.pedro.org.au, accessed on 14 September 2025).

## Data Availability

The data presented in this study are available on request from the corresponding author due to (specify the reason for the restriction).

## Abbreviations

The following abbreviations are used in this manuscript:

BMD	Bone mineral density
VRBT	Virtual reality-based therapy
NIVR	Non-immersive virtual reality
DLAs	Daily living activities
SRMA	Systematic review with meta-analysis
QoL	Quality of life
SMD	Standardized mean differences
95% CI	95% confidence interval
RCTs	Randomized controlled trials
